# Metastasis-directed stereotactic body radiotherapy for oligometastatic renal cell carcinoma: extent of tumor burden eradicated by radiotherapy

**DOI:** 10.1007/s00345-021-03742-1

**Published:** 2021-05-27

**Authors:** Yang Liu, Wen Long, Zhiling Zhang, Zitong Zhang, Lixin Mai, Sijuan Huang, Hui Han, Fangjian Zhou, Pei Dong, Liru He

**Affiliations:** 1 Department of Radiation Oncology, Sun Yat-Sen University Cancer Center, State Key Laboratory of Oncology in South China, Collaborative Innovation Center for Cancer Medicine, 651 Dongfeng Road East, Guangzhou, 510060 People’s Republic of China; 2 Department of Nuclear Medicine, Sun Yat-Sen University Cancer Center, State Key Laboratory of Oncology in South China, Collaborative Innovation Center for Cancer Medicine, 651 Dongfeng Road East, Guangzhou, 510060 People’s Republic of China; 3 Department of Urology, Sun Yat-Sen University Cancer Center, State Key Laboratory of Oncology in South China, Collaborative Innovation Center for Cancer Medicine, 651 Dongfeng Road East, Guangzhou, 510060 People’s Republic of China

**Keywords:** Carcinoma, renal cell, Radiosurgery, Stereotactic techniques, Metastasis

## Abstract

**Purpose:**

We aimed to explore whether complete eradication of tumor burden with stereotactic body radiotherapy (SBRT) would affect the outcomes of oligometastatic renal cell carcinoma (RCC).

**Materials and methods:**

Patients diagnosed with extracranial oligometastatic RCC (no more than five metastases) between 2007 and 2019 were reviewed. Those without nephrectomy were excluded. SBRT to all, some and no lesions were defined as complete, incomplete, and no SBRT. Progression-free survival (PFS) and cancer-specific survival (CSS) were analyzed using Kaplan–Meier method, Cox regression model and the Fine and Gray method.

**Result:**

A total of 101 patients were included, 51.5% of whom had < 3 metastases. Forty (39.6%) patients received complete SBRT, and 61 (60.4%) received no or incomplete SBRT. The 1-year LC rate was 97.3%. The complete SBRT group had significantly longer PFS (26.0 vs 18.8 months; *p* = 0.043) and CSS (not reached vs. 55.3 months; *p* = 0.012) compared with the no or incomplete SBRT group. In multivariate analysis, ECOG 0–1 (HR 0.389, 95% CI 0.167–0.906, *p* = 0.029) and complete SBRT were prognostic factors for CSS (HR 0.307, 95% CI 0.108–0.876, *p* = 0.027). Complete SBRT was associated with improved CSS in the subgroups of patients with age < 55 years, ECOG 0–1, clear-cell histology, IMDC intermediate/poor risk, metachronous metastasis, and < 3 lesions.

**Conclusion:**

Complete eradication of tumor burden with SBRT was associated with better survival in patients with oligometastatic RCC. The recommendation of SBRT to all lesions should be individualized.

**Supplementary Information:**

The online version contains supplementary material available at 10.1007/s00345-021-03742-1.

## Introduction

Renal cell carcinoma (RCC) accounts for 3%–5% of adult cancers [1]. Around 15% patients present with metastatic disease at diagnosis [2], and approximately 15–25% patients will eventually develop metastases after initial curative treatment [3, 4]. The oligometastatic paradigm suggests a stepwise process of acquiring metastatic ability. In the oligometastatic state, usually defined as limited number of metastases, metastasis-directed local therapy may delay or prolong the use of systemic therapy, or even cure some of the patients.

In oligometastatic RCC, the initial evidence supporting proactive local therapy is obtained from metastasectomy. A significantly longer survival (37–142 months) was observed after complete metastasectomy in comparison with incomplete or no metastasectomy (8–56 months) [5–7]. However, surgical complications, especially in the era of targeted therapy, is a common concern in these patients [8]. Stereotactic body radiotherapy (SBRT) is a non-invasive technique that could overcome the radioresistance of RCC by delivering intensified radiation doses per fraction. The meta-analysis of SABR ORCA demonstrates that SBRT could safely achieve a remarkable tumor control in oligometastatic RCC, with local control at around 90% and any significant toxicity at about 1% [9]. Given the non-inferior performance as compared with metastasectomy, SBRT has been endorsed as one of the first-line treatment for oligometastatic RCC in guidelines set forth by the National Comprehensive Cancer Network guidelines since 2019 [10].

Although numerous studies have confirmed the efficacy of SBRT in oligometastatic RCC, the percentage of patients receiving SBRT to all metastatic lesions varied. No study to date has evaluated whether the proportion of metastatic lesions eradicated by SBRT might affect the survival of patients with oligometastases. In this study, we aimed to explore the influence of eradicating all metastatic lesions with SBRT on survival in patients with oligometastatic RCC.

## Materials and methods

This retrospective study was performed under a waiver of informed consent approved by the Institutional Review Board (IRB number: B2020-056-01). Consecutive patients with extracranial metastatic RCC treated with targeted therapy at our institution between 2007 and 2019 were retrospectively reviewed. Inclusion criteria were patients with oligometastatic RCC, defined as no more than 5 lesions at the detection of metastatic disease [16]. Patients who did not receive nephrectomy were excluded. SBRT was recommended to oligometastatic patients with good ECOG performance status since 2015 in our institution, but the actual implementation of SBRT was affected by patients’ willingness to receive radiotherapy, their incomes and their insurances. SBRT delivered to all lesions was defined as complete SBRT, and SBRT directed at some of the lesions was defined as incomplete SBRT [30]. Patients receiving no SBRT were categorized as no SBRT.

For SBRT, all patients underwent 3 mm slice thickness contrast-enhanced computed tomography (CT) with site-specific immobilization. Motion assessment of lesions in lung and upper abdomen was realized by four-dimensional (4D) CT simulation scans. For lesions locating in bones or soft tissues, magnetic resonance imaging (MRI) simulation was usually implemented. Volumetric intensity modulated arc therapy techniques were used for treatment planning. All lesions were prescribed with ablative dose, that is, the maximum dose that could be achieved according to their vicinity to normal tissues, regardless of the indication of SBRT. Prescription dose was required to cover more than 90% of the target. Normal tissue dose constraints were in accordance with UK Consensus on Normal Tissue Dose Constraints for Stereotactic Radiotherapy and the Report of AAPM Task Group 101. Daily cone beam CT was carried out to verify patient position prior to treatment initiation. SBRT was executed once daily or every other day.

Patients were followed up every 3 months after the completion of SBRT. Clinical evaluation and imaging (usually CT scans) were acquired at each visit. MRI scans with contrast were recommended for bone metastases. Local control (LC) was calculated from SBRT to infield progression of irradiated sites. Progression-free survival (PFS) and cancer-specific survival (CSS) were measured from the diagnosis of oligometastasis to disease progression and cancer-related death. Adverse events were graded according to the Common Terminology Criteria for Adverse Events Version 5.0. Biologically effective dose (BED) was calculated using linear-quadratic model with *α*/*β* = 3 [11].

Chi-squared test was used for categorical data comparison, and Mann–Whitney tests were used for continuous variables. Survival was estimated by the Kaplan–Meier method and compared by log-rank test. Univariate and multivariate analyses were performed, and hazard ratio (HR) and associated 95% confidence intervals (CI) were evaluated by the cox proportional hazards model. Variables that were significant in the univariate analysis were included in the multivariate model. Competing risk analyses were also performed with the Fine and Gray method for both univariate and multivariate analyses for CSS, considering the death due to other causes. Statistical analysis was performed using SPSS version 23 (IBM Corp., Armonk, NY, USA) and R version 4.0.4, with two-sided statistical testing at the 0.05 significance level.

## Results

### Baseline characteristics

A total of 101 patients with oligometastatic RCC were identified. Baseline characteristics of patients are summarized in Table 1. The median number of metastatic lesions was 2 (1–5). The number of patients with 1, 2 and 3 organs involved at the time of metastasis were 86 (85.1%), 13 (12.9%), and 2 (2.0%), respectively.

**Table 1:** Baseline characteristics of the entire cohort (*N* = 101)

Characteristics	Overall (*N* = 101)	No or incomplete SBRT (*N* = 61)	Complete SBRT (*N* = 40)	*P*
Median age (range)	55 (18–86)	56 (18–86)	53 (20–77)	0.339
Sex				0.075
Male	73 (72.3)	48 (78.7)	25 (62.5)	
Female	28 (27.7)	13 (21.3)	15 (37.5)	
Histology				0.827
Clear cell	72 (71.3)	43 (70.5)	29 (72.5)	
Non-clear cell	29 (28.7)	18 (29.5)	11 (27.5)	
ECOG performance status				0.327
0–1	81 (80.2)	47 (77.0)	34 (85.0)	
> 1	20 (19.8)	14 (23.0)	6 (15.0)	
IMDC risk group				0.671
Favorable	25 (24.8)	16 (26.2)	9 (22.5)	
Intermediate/poor	76 (75.2)	45 (73.8)	31 (77.5)	
Synchronous metastasis				0.817
Yes	39 (38.6)	23 (37.7)	16 (40.0)	
No	62 (61.4)	38 (62.3)	24 (60.0)	
No. of lesions				0.073
1–2	52 (51.5)	27 (44.3)	25 (62.5)	
3–5	49 (48.5)	34 (55.7)	15 (37.5)	
Organs involved				0.092
Single	86 (85.1)	49 (80.3)	37 (92.5)	
Multiple	15 (14.9)	12 (19.7)	3 (7.5)	
Metastasectomy	19 (18.8)	17 (27.9)	2 (5.0)	0.004

All patients were treated with targeted therapy. Tyrosine kinase inhibitors were the most commonly chosen, accounting for 97.0% cases. The number of patients receiving sorafenib, sunitinib, axitinib, and pazopanib were 12 (11.9%), 56 (55.4%), 21 (20.8%), and 8 (7.9%), respectively. Among the patients receiving SBRT, systemic therapies were initiated after SBRT in 5 patients (9.8%) around 0.7 months after SBRT. Three of them (60.0%) received sunitinib, and the rest (40.0%) received axitinib. The remaining patients started systemic therapy before SBRT.

Forty (39.6%), 11 (10.9%), and 50 (49.1%) patients received complete, incomplete, and no SBRT. Baseline characteristics including age, sex, performance status, IMDC risk group, number of metastases, and synchronous metastasis were similar between the complete SBRT and no or incomplete SBRT group. Among the 46 patients receiving systemic therapy before SBRT, the rates of partial response or stable disease before radiotherapy were similar between the complete and incomplete SBRT group (77.1 vs 63.6%, *p* = 0.620).

### Radiation dose and local control

SBRT was performed in a total of 114 lesions (Supplementary Table S1). The number of patients receiving SBRT to 1, 2, 3, 4, and 5 lesions were 19, 15, 7, 6, and 4, respectively. Twenty-five (45.5%) patients were symptomatic at the time of SBRT. The most common fractionation scheme was 30–45 Gy in 5 fractions, accounting for 107 sites (93.9%). The median BED was 147 Gy (range, 88–180 Gy) for the complete SBRT group, and 147 Gy (range, 117–187 Gy) for the incomplete SBRT group. Two lesions developed infield recurrence after SBRT, occurring in adrenal gland and thoracic vertebra. Both of these lesions presented with initial partial response followed by infield disease progression 11.0 and 11.7 months after SBRT. The 1-year LC rate by site was 97.3%.

### Survival outcomes and prognostic factors

**Fig. 1:**
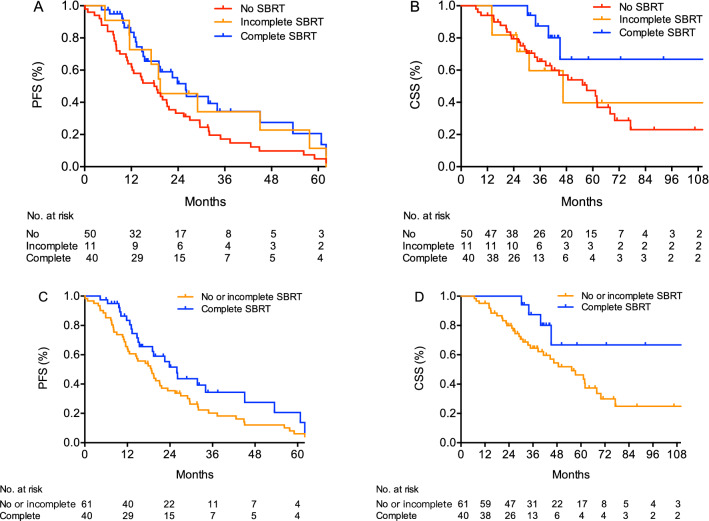
Progression-free survival **A** and cancer-specific survival **B** of patients receiving different extent of tumor burden eradicated by SBRT. Progression-free survival **C** and cancer-specific survival **D** of patients treated with and without complete SBRT

The median follow-up for the entire cohort was 31.0 months (range, 6.0–133.0 months). Thirty-seven cancer-related deaths (36.6%) were observed. Four patients (4.0%) were lost to follow-up. The median PFS and CSS of the entire cohort was 19.5 months and 61.2 months, respectively. The median PFS of patients in the complete, incomplete, and no SBRT group were 26.0 months, 19.4 months, and 17.9 months, respectively (*p* = 0.049) (Fig. 1A). The 2 year CSS for patients receiving complete, incomplete, and no SBRT were 100, 81.8, and 79.5%, respectively (*p* = 0.044) (Fig. 1B). The group of patients treated with complete SBRT had significantly longer PFS compared with those with no or incomplete SBRT (26.0 vs 18.8 months; *p* = 0.043) (Fig. 1C). The median CSS of the complete SBRT group was not reached (NR), compared with 55.3 months in the no or incomplete SBRT group (*p* = 0.012) (Fig. 1D).

In univariate analysis, better CSS was observed in patients with ECOG performance status 0–1, IMDC favorable risk, and complete SBRT (Supplementary Table S2). Age, histological type, synchronous metastasis, the number of lesions, the number of organs involved, and metastasectomy were not significant prognostic factors for CSS. After including ECOG performance status, IMDC risk group, and the extent of SBRT in multivariate analysis, ECOG 0–1 (HR 0.389, 95% CI 0.167–0.906, *p* = 0.029) was independently associated with superior CSS, and complete SBRT demonstrated a significant decreased risk of cancer-related death by 69% (HR 0.307, 95% CI 0.108–0.876, *p* = 0.027).

**Fig. 2:**
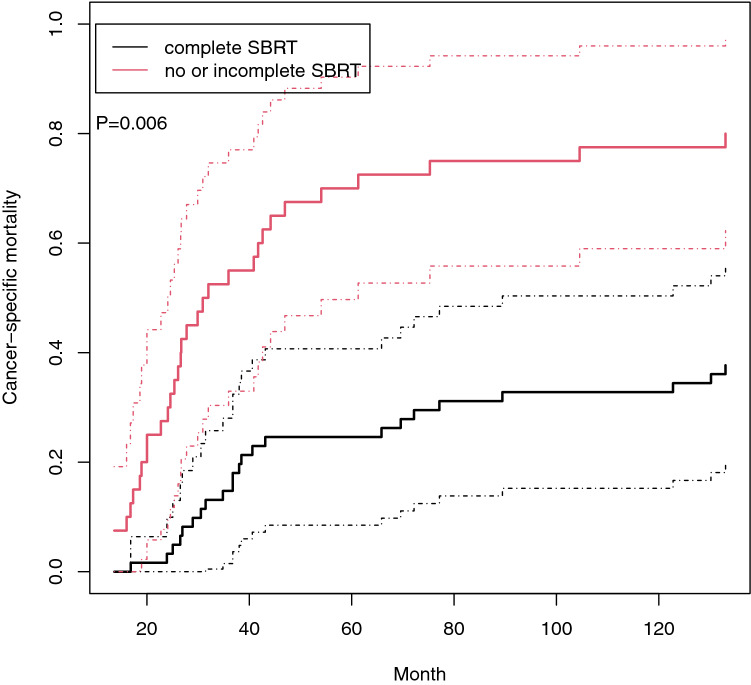
Cancer-specific mortality of the patients with and without complete SBRT using Fine and Gray competing risk analysis

In the competing risk analyses using Fine and Gray method, complete SBRT (*p* = 0.006) (Fig. 2) and the number of lesions (*p* = 0.036) were significant prognostic factors for CSS. Age, histological type, ECOG performance status, IMDC risk group, synchronous metastasis, the number of organs involved, and metastasectomy were not significant prognostic factors. In multivariate analysis, only complete SBRT remained significant (HR 0.273, 95% CI 0.097–0.765, *p* = 0.014).

### Subgroup analyses for complete SBRT

To identify potential candidate for complete SBRT, subgroup analyses were performed. Compared with incomplete or no SBRT, complete SBRT was associated with improved CSS among patients with age < 55 years, metachronous metastasis, and < 3 lesions (Supplementary Figure S1). ECOG performance status, histology, IMDC risk groups, and number of organs involved were not analyzed due to the small sample size in certain subgroups. The median CSS of patients receiving complete SBRT was not reached, compared with 46.4 months of those receiving no or incomplete SBRT in the subgroup with < 3 metastases (*p* = 0.009). In patients with ≥ 3 metastases, no significant difference of CSS between subgroups was found (*p* = 0.443) (Supplementary Figure S2).

### SBRT-related toxicity

SBRT was generally well tolerated. No grade 4 or 5 toxicity was observed. There were 1 case of grade 3 toxicity and 16 cases of grade 2 toxicity in 16 patients (15.8%) (Supplementary Table S3). Among the bone lesions treated with SBRT, 12 cases of fracture were observed, most of which were grade 1 or 2. One patient suffered from progressively worsening left thigh pain following SBRT to the L4 vertebra due to spinal compression fracture with the nerve root compression. The patient underwent vertebroplasty and biopsy of the irradiated site 7 months after SBRT. No residual cancer was found on biopsy.

## Discussion

Metastasis-directed therapy with SBRT is safe and efficacious in the management of patients with limited metastatic burden, but the influence of tumor burden eradicated on clinical outcome remains undiscussed. In our study, we showed that patients with all metastases eradicated by SBRT had significantly improved CSS. Complete SBRT was associated with longer CSS in patients with metachronous disease, and < 3 metastatic lesions.

Numerous studies on metastasis-directed surgery repeatedly point out that the extent of metastasectomy may affect the survival of RCC patients. A meta-analysis including 2267 patients reported that the overall survival ranged from 37 to 142 months for patients receiving complete metastasectomy, and 8–27 months for incomplete or no metastasectomy. The survival benefit from complete metastasectomy was observed across different studies, with a pooled HR of 2.37 [12]. Metastasis-directed therapy to all metastatic sites could eliminate resistant clones, minimize the chance of de novo driver somatic alterations of metastatic clones, and prevent metastasis-to-metastasis seeding [13]. Among all the locally ablative techniques, irradiation may provide additional gains through fulminant vascular damage and the activation of immune system [14]. A couple of studies shed light upon the value of SBRT in oligometastatic RCC, but the percentage of patients undertaking SBRT to all metastases were variable (32–100%) (Table 2) [15–19]. Compared with radiation to a single lesion, multisite irradiation could amplify the benefit of SBRT by maximally eradicating existing clones and enhancing the release and uptake of tumor-associated antigens [20, 31]. Our study revealed that the extent of tumor burden eradicated by SBRT affected the CSS of patients with oligometastatic RCC, comparable to the findings in surgical series [5, 21–23]. Our results suggest applying SBRT to all metastatic sites. Notably, in the era of immunotherapy, the results might be different, especially for the IMDC intermediate and poor risk group, which may be more in need of intensified systemic therapies.

**Table 2:** Summary of published literature on stereotactic body radiotherapy for oligometastatic renal cell carcinoma

Author, year	N	Median No. of lesions (range)	Metastatic sites	Synchronous metastasis (%)	Clear-cell type (%)	Lung (%)	Bone (%)	Complete SBRT (%)	Survival outcomes
Ranck, 2013 [15]	18	2	Bone, lymph node, lung	/	78	22	61	67	2y-OS 85% mPFS 12.7 m, 2y-PFS 36%
Meyer, 2018 [8]*	80	2 (1–5)	Brain, bone	39	85	/	32	100	mOS 33.9 m mPFS 7.6 m, mTTS 14.2 m
Stenman, 2018 [17]*	57	2	Lung, Bone, lymph node	49	86	24	59	32	Entire group: mOS 40 m; Complete SBRT group: mOS 51 m
Franzese, 2019 [18]	58	1 (1–3)	Lung, lymph node, bone	21	83	53	10	100	2y-OS 100%, 5y-OS 83% mPFS 11.1 m
Zhang, 2020 [19]	47	1 (1–4)	Bone, lung, liver, soft tissue	28	87	15	43	100	1y-OS 93%, 2y-OS 85% mFST 15.2 m

*Oligometastasis treated with stereotactic body radiotherapy represents a subgroup of patients in this study. *OS* overall survival, *PFS* progression-free survival, *FST* freedom from systemic therapy, *TTS* time to start or change of systemic therapy

The rationale behind local therapy in the metastatic setting is that an intermediate state with low, slow and late spreading capacity exists, generally known as oligometastasis. In metastatic RCC, smaller number of metastases and fewer organ sites involved were associated with better outcomes [22–24], while multiple lesions within one organ were associated increasing chance of progression following local therapy [25], implying that the number of metastases may aid in the selection of local therapy. In the study by Kwak et al., significantly prolonged overall survival was shown after metastasectomy in patients with solitary metastasis, and those with multiple metastases could not benefit from aggressive local resection [7]. Similarly, complete eradication of metastases by SBRT was associated with survival improvement in patients with < 3 metastases, but failed to demonstrate benefit in the subgroup of ≥ 3 metastases in our study. These findings suggest that although the number of metastases could not fully define oligometastasis, it is still a simple and intuitive way to identify candidates for local therapy at present.

In addition to quantitative elements, temporal factors may also reflect the biological behavior of RCC. Short disease-free interval to metastasis is recognized as an unfavorable prognostic factor [26]. Synchronous metastasis is associated with more aggressive behavior, such as advanced stage of primary site, higher proportion of sarcomatoid component, overexpression of vascular endothelial growth factor and PD-L1 [27]. Estimates indicate that curative metastasectomy could be yielded in 25% paitents with metachronous metastasis [28], and the rate may decline to less than 10% in the synchronous setting [29]. The TRACERx Renal study revealed that patients with synchronous metastasis might harbor higher genome instability that leads to the acquisition of metastatic ability early in the course of disease compared with the cases of metachronous metastasis [13]. Thus, metachronous metastasis is more likely be associated with indolent disease, in which metastasis-directed local therapy may provide survival benefit. In our study, complete SBRT was associated with improved CSS among patients with metachronous metastasis but not in those with synchronous metastasis, which imply that the timing of oligometastasis should also be considered before choosing cytoreductive local therapy.

Several aspects limit the generalizability of our study. First of all, the retrospective design with limited time of follow-up is an inherent shortcoming. Second, although the study cohort was predominantly treated with first-line tyrosine kinase inhibitors, we could not strictly control the type and timing of systemic therapies. Third, although we compared all the available baseline characteristics, these parameters may not fully represent the clinicopathological behavior of oligometastatic RCC. Lastly, SBRT was recommended to patients that could tolerate by the multidisciplinary team. Although the baseline performance status was similar between the two treatment groups, we could not fully rule out its influence on outcomes.

## Conclusions

Our study indicates that the extent of tumor burden eradicated by SBRT can affect the prognosis of patients with oligometastatic RCC. Complete eradication of metastatic sites with SBRT and performance status were prognostic factors for CSS. The recommendation of SBRT to all lesions should be individualized, and validation of these results by future prospective and randomized studies is needed.

## Supplementary Information

Below is the link to the electronic supplementary material.

Supplementary file1 (DOCX 228 KB)

## Data Availability

The data that support the findings of this study are available from the corresponding author upon reasonable request.
