# Supplementary material for: Metastasis-directed stereotactic body radiotherapy for oligometastatic renal cell carcinoma: extent of tumor burden eradicated by radiotherapy
Source: World J Urol. 2021 May 27;39(11):4183–90. doi: 10.1007/s00345-021-03742-1 (PMC8571216; doi:10.1007/s00345-021-03742-1)
Supplement: Supplementary file 1 — Supplementary file1 (DOCX 228 KB) [file 345_2021_3742_MOESM1_ESM.docx]

**Supplementary Materials**

Table S1. Dose and fraction of metastatic lesions (N = 114)

| Location | Sites (%) | Fractions | Median total dose, Gy (range) |
| --- | --- | --- | --- |
| Bone (N=58) | 2 (1.8) | 1 | 15.5 (15-16) |
|  | 2 (1.8) | 3 | 27 (24-30) |
|  | 54 (47.4) | 5 | 40 (35-45) |
| Lymph nodes (N=30) | 29 (25.4) | 5 | 35 (30-40) |
|  | 1 (0.9) | 6 | 36 |
| Lung (N=5) | 4 (3.5) | 5 | 45 (40-45) |
|  | 1 (0.9) | 8 | 56 |
| Adrenal gland (N=5) | 5 (4.4) | 5 | 42 (35-45) |
| Others (N=16) | 1 (0.9) | 4 | 36 |
|  | 15 (13.2) | 5 | 40 (35-43) |

Table S2. Prognostic factors for CSS (N=101)

| Variables | Univariate analysis | | Multivariate analysis | |
| --- | --- | --- | --- | --- |
|  | HR (95% CI) | P | HR (95% CI) | P |
| Age |  | 0.534 |  |  |
| ≥55 yrs vs <55 yrs | 0.814 (0.426-1.557) |  |  |  |
| Histology |  | 0.543 |  |  |
| Non-clear cell vs Clear cell | 1.245 (0.614-2.526) |  |  |  |
| Synchronous metastasis |  | 0.503 |  |  |
| Yes vs No | 0.786 (0.388, 1.592) |  |  |  |
| ECOG performance status |  | 0.002 |  | 0.029 |
| 0-1 vs >1 | 0.275 (0.121-0.624) |  | 0.389 (0.167-0.906) |  |
| IMDC criteria |  | 0.032 |  | 0.082 |
| Intermediate/poor vs Favorable | 2.484 (1.080-5.709) |  | 2.151 (0.908-5.095) |  |
| No. of metastatic lesions |  | 0.750 |  |  |
| 3-5 vs 1-2 | 1.112 (0.578-2.140) |  |  |  |
| Organs involved |  | 0.206 |  |  |
| Singe vs Multiple | 0.585 (0.255-1.342) |  |  |  |
| Metastasectomy |  | 0.380 |  |  |
| Yes vs No | 1.373 (0.676-2.788) |  |  |  |
| Extent of tumor burden removed |  | 0.019 |  | 0.027 |
| Complete vs No or incomplete SBRT | 0.287 (0.101-0.813) |  | 0.307 (0.108-0.876) |  |

Table S3. SBRT-related toxicity

|  | Grade 1 | Grade 2 | Grade 3 |
| --- | --- | --- | --- |
| Skin | 3 | 1 |  |
| Fatigue |  | 4 |  |
| Nausea/Vomiting | 13 | 2 |  |
| Duodenal hemorrhage | 1 |  |  |
| Neuropathy | 3 | 2 |  |
| Radiation pneumonitis | 6 |  |  |
| Fracture | 4 | 7 | 1 |
| Dysuria | 1 |  |  |

Figure S1. Forest plot of subgroup analysis for cancer-specific survival.

Figure S2. Cancer-specific survival of patients receiving complete SBRT versus no or incomplete SBRT in the subgroups of <3 metastases (A) and ≥3 metastases (B).

Figure S3. Swimmer plot of PFS of patients receiving complete and incomplete SBRT.
